# Survival, trust, and compassion: persistent HIV prevention concerns faced by Black sexual and gender minority communities in the US South – a qualitative study

**DOI:** 10.3389/fpubh.2025.1695474

**Published:** 2025-11-14

**Authors:** Jesse Strunk Elkins, Ian Dale, Eunice Okumu, Matthew Zinck, Carol Golin, Jordyn McCrimmon, Maria Esposito, Isabelle T. Duguid, Dalton M. Craven, Ujunwa Onyeama, Sebastian Marin-Cespedes, Alicia Diggs, Marielle Indyg, Ann M. Dennis, Meagan Zarwell

**Affiliations:** 1 Department of Epidemiology and Community Health, College of Health and Human Services, UNC Charlotte, Charlotte, NC, United States; 2 Division of Infectious Diseases, Department of Medicine, UNC-Chapel Hill School of Medicine, Chapel Hill, NC, United States; 3 Center for AIDS Research, UNC-Chapel Hill, Chapel Hill, NC, United States; 4 Department of Health Behavior, Gillings School of Global Public Health, UNC-Chapel Hill, Chapel Hill, NC, United States; 5 Division of General Internal Medicine and Clinical Epidemiology, Department of Medicine, UNC-Chapel Hill School of Medicine, UNC-Chapel Hill, Chapel Hill, NC, United States; 6 Department of Psychology, College of Humanities and Social Sciences, North Carolina State University, Raleigh, NC, United States; 7 Violence Prevention Center, UNC Charlotte, Charlotte, NC, United States

**Keywords:** HIV, PrEP, sexual and gender minority health, social network strategy, prevention

## Abstract

**Background:**

Members of Black sexual and gender minority (BSGM) groups in the Southern US experience disparities across all pillars of the HIV prevention continuum. Social network strategy (SNS) is an intervention that trains people with reasons to test for HIV to reach out to peers to increase HIV testing and may improve uptake of pre-exposure prophylaxis (PrEP). We conducted formative research to design an enhanced SNS to implement within partner services.

**Methods:**

In 2022–2024, we conducted four focus groups among HIV public health services staff and 12 in-depth interviews with local health department officials, clinicians, and community-based organization leaders. In addition, we interviewed four BSGM community members who recently used prevention services (i.e., testing or PrEP). Focus groups and interviews were transcribed and iteratively coded. Themes included salient barriers and facilitators to HIV services and mapped to a modified socio-ecological model after analysis. Narratives from the four BSGM were integrated with the summary analyses from HIV service providers.

**Results:**

Service providers and community members identified persistent barriers to HIV prevention services: (1) basic human needs for survival and (2) lack of compassion within the healthcare system. Inadequate living necessities (e.g., housing and transportation), education, mistrust, stigma, and discrimination limit HIV prevention service use. Conversely, wrap-around services, transparency, representation, and compassionate care facilitate service access.

**Conclusion:**

Enhancing HIV prevention services for BSGM groups necessitates reducing healthcare access barriers and leveraging facilitators. Our findings informed an enhanced SNS intervention to increase HIV testing, prevention, and care linkage among BSGM in the Southern US.

## Background

The ‘Ending the HIV Epidemic’ (EHE) US policy identified 57 priority jurisdictions, many in the US South, that account for more than 50% of all new HIV diagnoses in the US (1). Mecklenburg County, North Carolina (NC), and the neighboring state of South Carolina are two priority jurisdictions where interventions are necessary to reduce frequent HIV transmissions. Black sexual and gender minority (BSGM) communities, including men who have sex with men and transgender women, consistently face disproportionate and worsening health inequities (e.g., social stigma, health insurance coverage, and inadequate education), with the greatest number of new HIV diagnoses occurring within these groups (1–4). Such disparities across the HIV prevention continuum result in higher rates of HIV incidence and lower engagement in prevention services like pre-exposure prophylaxis (PrEP) (5–7).

Additionally, BSGM face a constellation of challenges within their social networks, (e.g., social, sexual, and geographic proximity to networks with greater incidence of HIV) that contribute to the urgent need to increase PrEP utilization among BSGM priority areas in the US South (8–10). BSGM face significantly higher levels of multiple marginalization, social stigmas, and discrimination, which increase their vulnerability to HIV exposure compared to non-BSGM groups (11). Data from the 2014–2018 Behavioral Risk Factor Surveillance System examined the intersectionality of being Black in the US and identifying as a gender minority (such as transfeminine, transmasculine, or another gender-diverse identity). The findings revealed that BSGM are disproportionately affected by race and gender intersectionality, thus experience worsening forms of oppression (11). BSGM are more likely than cisgender Black individuals and gender minority white individuals to self-report recent injection drug use, exposure to sexually transmitted infections, history of sex work, condomless sex, and more than four sex partners yearly (11). Higher rates of HIV-related stigma have been reported in the US South, where social conservatism and stigmatizing faith beliefs persist (2, 12, 13). Stigma, medical mistrust, geographic accessibility, lack of health insurance, financial assistance programs, and insufficient HIV awareness are universal barriers to prevention and care engagement (4). Compared to other regions, Southern states report greater religiosity, lower levels of HIV-related public health funding, and fewer nondiscrimination protections for sexual and gender minorities (2, 14, 15). These factors contribute to heightened stigma, medical mistrust, and limited access to culturally competent care, further increasing HIV risk factors (16–18). Analyses of national survey data reveal that rural identity, which is disproportionately represented in the South, predicts less supportive attitudes toward LGBTQ people and policies; and peer-reviewed syntheses and Southern samples document persistently lower social acceptance and fewer protections in the South than in the Northeast or West (19, 20). These cultural and structural differences make the South a critical region for studying BSGM experiences of HIV prevention and care engagement.

Improving these systemic disparities is critical to reducing health disparities among BSGM (3). In addition, social capital within BSGM networks may impact the likelihood of HIV transmission by increasing awareness and acceptability of PrEP, especially among those who experience multiple forms of marginalization (3). Social network approaches for HIV prevention leverage existing network relationships and network structures to promote behavior changes (i.e., increased HIV testing and PrEP uptake) (21). Just as exposures or behaviors can cluster within networks, beneficial health information can also cluster in networks, promoting healthier behavioral norms (22).

The CDC’s Social Network Strategy (SNS) is an evidence-supported intervention designed to motivate individuals from disproportionately affected groups, such as BSGM, to test for HIV (23–26). SNS enlists people living with HIV or those with a greater chance of acquiring HIV to recruit peers for HIV testing based on relationships with shared social similarities (27). Demonstration projects have shown SNS to be effective in increasing HIV testing among BSGM, including in the US South (25, 28). The present study aims to build upon understandings of SNS by identifying lived experiences with HIV prevention services among BSGM communities and service providers. Public health professionals, advocates, and members of BSGM networks shared their experiences through focus groups and in-depth interviews, describing barriers and facilitators to HIV prevention services.

## Methods

### Design and setting

This cross-sectional qualitative study explored potential barriers and facilitators to HIV prevention services for BSGM from the perspective of two participant groups: leadership participants and BSGM community members. This study will report on barriers and facilitators to HIV service utilization to inform the development and implementation of enhanced Social Network Strategy (eSNS) in response to identified needs of BSGM in recent transmission networks. This methodical response aims to increase HIV service utilization in Charlotte, NC for BSGM. This study was approved by the Institutional Review Board (IRB) at the University of North Carolina Chapel Hill.

This study was guided by an interpretivist epistemology, which views knowledge as co-constructed through lived experience, relationship, and context rather than discovered as objective fact. We approached the research with the understanding that participants’ narratives reflect situated truths shaped by their intersecting identities as BSGM individuals living in the US South. Within this framework, meaning emerges through dialog, reflexivity, and embodied understanding, recognizing that experiences of trust, stigma, and compassion are both personal and socially produced. The research team engaged in ongoing reflexive practice to acknowledge how our identities, disciplines, and proximity to the work influenced interpretation. This stance allowed us to center BSGM participants’ meaning-making processes and to interpret their stories as expressions of resilience, survival, and care within systems that have historically marginalized them.

Our interdisciplinary team included public health professionals, physicians, and social scientists from UNC Chapel Hill and UNC Charlotte with diverse identities across race, gender, sexuality, age, and geography. Two team members identify as Black women, one of whom is Kenyan-born and has lived in the US South for nearly two decades; others identify as white, with one genderfluid and several queer or non-heterosexual members. While most team members are outsiders to the lived experiences of BSGM communities and to living with HIV, all share commitments to equity, compassion, and community-engaged research. We approached analysis reflexively, using paired coding across institutions, memoing, and regular discussion to examine how our positionalities shaped interpretation. Black and queer team members provided critical insight into intersectional stigma, helping redirect analysis toward structural accountability rather than individual deficit. None of the interviewers had prior personal relationships with participants. Throughout the study, reflexive dialog emphasized humility and an intent to center participants’ voices and lived expertise.

### Data collection

#### Participants

We purposively recruited HIV leadership participants (i.e., public health professionals and advocates) to participate in in-depth interviews (IDIs) and focus groups discussions (FGDs). Participants were recruited through the investigators’ networks by email invitation to county health department leadership and local community-based organizations (CBO), and clinics. Additionally, we recruited BSGM community members (i.e., local community members in sexual contact with someone recently diagnosed with HIV, who received routine partner services like HIV testing) to participate in IDIs. Eligible participants were sent an electronic consent form to read and sign before participating. Leadership FGDs and IDIs were conducted from December 2022 to February 2023. BSGM community member IDIs were conducted from June 2023 to February 2024. FGDs and IDIs were conducted over Zoom by interviewers and audio recorded.

To ensure rigor, all interviewers reviewed the interview guides with the analysis team and practiced one mock interview with a member of the study team prior to conducting participant interviews. All IDI interviewers reviewed the interview guides with the analysis team and each one practiced one mock IDI with a member of the study team prior to conducting participant interviews. The two staff members who facilitated the FGDs have close to 20 and 10 years of experience in qualitative research respectively, including in data collection. Team members attended data collection trainings, including formal Qualitative Research trainings. The RESPOND-study specific training included first reading, aloud as a group, the study protocol, ICFs, data collection guides and data collection SOP, followed by conducting mock interviews. Two FGD mock interviews were conducted facilitated by the two most experienced qualitative researchers. Each team member also conducted one mock interview per IDI activity (i.e., Leadership IDIs and BSGM IDIs) (see Table 1).

**Table 1:** Leadership and BSGM community member characteristics.

Participants		%	*n*/*N*
In-depth interviews with public health leaders (*N* = 12)			
Gender	Female	66.7%	8/12
	Male	33.3%	4/12
Age	Median (range)	47	(28–66)
Race	White	58.3%	7/12
	African American/Black	41.6%	5/12
Ethnicity	Non-Hispanic/Latino	91.6%	11/12
	Hispanic/Latino	8.3%	1/12
Position title	Executive	41.7%	5/12
	Program Manager	33.3%	4/12
	Medical Director	25%	3/12
Practicing clinician	No	66.7%	8/12
	Yes	33.3%	4/12
Time in current role (years)	Median (range)	3.5	(1–13)
Professional experience (years)	Median (range)	18	(10–40)
Focus group interviews (*N* = 19)			
Position title	Case manager	47.3%	9/19
	Public health investigator	42.1%	8/19
	Program manager	5.3%	1/19
	Unknown	5.3%	1/19
Time in current role (years)	Median (range)	1.5	(<1–4)
Professional experience (years)	Median (range)	13	(4–25)
In-depth interviews with men who were not living with HIV (*N* = 4)			
Age	Median (range)	33.5	(24–39)
Race	African American/Black	100%	4/4
Ethnicity	Non-Hispanic/Latino	100%	4/4

Finally, IDI’s were conducted with leadership who do not necessarily perform partner services. FGDs were conducted with DIS and other folks involved in partner services who could possibly implement eSNS. Given the power differentials, IDIs with leadership were required. FGDs shared more common work responsibilities and experiences.

#### Leadership focus group discussions (FGDs)

Four FGDs, which each lasted approximately 120 min, were comprised of 4–6 leadership participants. In total, 19 staff from the local health department or CBOs who provide direct services to clients participated in FGDs 9 case managers, 8 HIV/STI disease intervention specialists (DIS)/public health field investigators, and 2 supervisors of partner services and/or linkage to care programs. Three of the four FGDs included at least one CBO staff member; participants’ time in their current roles ranged from less than 1 to 15 years. FGDs occurred during regular work hours and were not compensated. Interviewers practiced with two mock FGDs (i.e., one per facilitator) with one note-taker and at least three other team members.

#### Leadership and BSGM community member in-depth interviews (IDIs)

IDIs were conducted with 12 participants in leadership roles (5 public health departments, 4 CBOs, and 3 local clinics). Additionally, four BSGM community member IDIs were conducted with BSGM participants, all of whom were not living with HIV and identified as cisgender men. Interviews lasted approximately 60 min. Interview participants were given a $50 gift card for their time.

#### Interview guides

The IDI and FGD guides for leadership participants explored how they perceived BGSM groups’ engagement in local prevention services, barriers to access, and factors that could enhance engagement. Additionally, we aimed to understand effective methods for peer recruitment for HIV testing and PrEP engagement. IDI guides for BSGM community members were informed by theories of social networks and health, and we considered how social networks, shaped by socio-geographical factors, may influence behaviors and ultimately impact group and population outcomes (29). Additionally, we explored the influence of social and interpersonal behaviors, like social support, social influence, and access to various resources (30). Questions from the FGD and IDI guides are provided in Supplementary Tables S1, S2.

### Data analysis

Audio-recorded FGDs and IDIs were transcribed using Landmark transcription services. Transcripts were initially coded using NVivo 1.7 by QSR International and then uploaded to Atlas. Ti to make coded data accessible to team members across institutions. Leadership data were collected from December 2022 to February 2023 (FGDs in December 2022 and IDIs through February 2023), while BSGM community member data were collected from June 2023 to February 2024, with data analysis conducted sequentially and formal analyses continuing through November 2024. Recruitment continued until data saturation was achieved, as indicated by the repetition of prior findings in transcripts and debriefs. FGDs were not stratified, and data were monitored using constant comparison during data collection to assess general and across-group saturation on core HIV prevention topics (i.e., testing and PrEP). Saturation for FGDs was determined through an emergent design, as participants consistently identified overlapping barriers and facilitators to eSNS across discussions. The team used Cohen’s Kappa scores to facilitate discussion to improve consensus of the codebook application. During coding meetings, the team discussed agreement rates below 80% to reconcile discrepancies in coding and make iterative modifications to the codebook to improve coding consistency. The final codebook included inductive and deductive codes and was applied to leadership FGD and IDI transcripts. Two separate groups of coders independently coded one FGD and one IDI transcript, performed inter-rater reliability checks in NVivo, and resolved any discrepancies before coding the remaining transcripts. For the BSGM community member IDI analysis, three pairs of coders applied inductive codes using a Modified Social Ecological Model (MSEM) iteratively.

Thematic analysis of IDIs and FGDs was guided by Braun and Clarke’s approach (31) through November of 2024. The coded data were reviewed in framework matrices characterizing perceptions about engagement in HIV prevention services among BSGM in Charlotte, NC and sorted into potential themes and sub-themes, discussed among the study team, and mapped onto each level of the MSEM Framework (e.g., individual, social network, community, and policy). Analysts grouped data coded with HIV prevention into potential barriers and facilitators, discussed emerging themes, and iteratively drafted analytical memos to enhance credibility. Results were refined and organized into two reports, one for each participant group. Subsequently, JSE and MCZ developed overarching themes across data sources to integrate results from providers with personal experiences shared by BSGM community members. We modified quotations for conciseness by removing pauses or repeated phrases.

## Results

Participant demographics are displayed in Table 1. Among the 12 individuals from the IDIs with public health leaders, the majority were non-Hispanic (91.6%), White (58.3%), and Female (66.7%), with a median age of 47 years (range: 28–66). All four participants from the IDIs with men who were not living with HIV were non-Hispanic and African American/Black, with a median age of 33.5 years (range: 24–39).

### Overview of barriers to HIV prevention services from leadership participants

Leadership participants described HIV testing and PrEP access barriers for BSGM. Overarching barriers to HIV prevention services reported in both leadership FGDs and IDIs included: (1) lack of HIV prevention/PrEP knowledge; (2) confidentiality/disclosure concerns; (3) socio-economic obstacles; (4) HIV self “risk” perceptions; (5) PrEP inaccessibility; (6) experienced PrEP burden; (7) experienced stigma; (8) unclear PrEP campaign messaging (e.g., undefined access options); and (9) lack of provider trust. HIV testing results wait-time, PrEP efficacy, and PrEP modalities were other recurring barriers discussed in IDIs only. Additional barriers mentioned in the IDIs included mental health, fear of needles, lack of representation in health care facilities and staffing issues.

### Overview of facilitators to HIV prevention services from leadership participants

Overarching facilitators reported in both leadership FGDs and IDIs included: (1) PrEP education; (2) PrEP accessibility; (3) PrEP modality options; (4) PrEP campaign messaging; and (5) trust in providers. Confidentiality/disclosure and socio-economic factors were other recurring themes reported in the IDIs only, while PrEP efficacy was reported in the FGDs only. Additional facilitators reported in the IDIs only included sexual health education and HIV self-testing/mobile testing, while fewer PrEP side effects were reported as an issue in the FGDs only.

Using these overarching barriers and facilitators provided by leadership participants, we describe themes that were also highlighted and reinforced by stories shared by four BSGM community member interviews (three of whom were taking PrEP). Below, we define two central themes that collectively represent dominating barriers and facilitators described in leadership participant FGDs and IDIs, and BSGM community member IDIs: (1) How do We Navigate and Negotiate PrEP When the Options are “Survival” vs. “Health”? (see Tables 2, 3), and (2) Compassion from Others is the Antidote to Mistrust When We Do not Know Who to Believe (see Tables 4, 5).

**Table 2:** BSGM community member perspectives: Navigating and Negotiating PrEP: “Survival” vs. “Health.”

Summary	Quote
Participant A discusses the struggle of “living paycheck to paycheck.”	Participant A “Um, you know, I-I live paycheck to paycheck. Um…. So, you know, right now we kinda—you know, diggin’ through the back of the refrigerator trying to make some food that is not real food until, uh, Thursday, um… when I get paid. And that’s not-it’s not always my situation, but it’s been, um, periodically throughout my adult life, that’s been something I’ve had challenge of.” (Cis man, taking PrEP, 36 years).
Participant B notes the employment discrimination that transgender women often face and the challenges of securing or keeping a job due to biases related to gender identity.	Participant B “I mean, I’m pretty sure it’s hard for [transgender women] to probably find a job, I mean, or to keep a job. I mean, once they-I mean, once somebody probably see them come in as a woman, thinkin’ that it’s a woman, then they look at their resume or look at their profile and everything and it says that they was a male at birth, you know—and someone can discover that against ‘em, you know?… Just because they dress a certain way, that should not stop them from gettin’ a job.” (Cis man, taking PrEP, 39 years).
Participant C highlight barriers to PrEP access for BSGM with emphasis on financial, educational, and locational challenges.	Participant C “Making sure that they have, um, access from a financial standpoint of view, education or exposure to, um, resources or individuals who are willing to talk to them and kinda meet them where they are at… and then making sure that the care they have is within, you know, not too far a drive away… Yeah, I think definitely employment services, um, and definitely access to food, I think are two.” (Cis man, taking PrEP, 35 years). “I think-I think most of it comes down to—in my opinion, comes down to like education… Uh, just letting people, you know, exposure and letting people know these programs are out there.” (Cis man, taking PrEP, 35 years).
Participant D highlights the challenge of maintaining privacy when receiving PrEP.	Participant D “I did not want like my packages to arrive I ordered through a third party attempting to receive my PrEP. And I did not want it just pop up with my information and everything on it. So, I had—my thing was finding a discreet way for me to receive the drugs.” (Cis man taking PrEP, 24 years).

**Table 3:** Leadership perspectives: Navigating and Negotiating PrEP: “Survival” vs. “Health.”

Barriers	
Basic needs	“I think also just, like, going back to kind of, like, the bare necessities, like housing and food security, um, so obviously, if they do not have those things, I mean, getting tested or getting into care or staying into care is not a top priority […] just that even if they do have, like, even if they are not already homeless, like, they already have secured housing. Like, it’s not always that secure…” (FGD3, Public Health Investigator).
Access to HIV/STI information	“Well, where do I go to access this? Like, ‘who-who do I talk to?’ or— ‘who do I get connected with?’ and making sure that they have someone to reach to say, ‘hey. I wanna get on PrEP.’” (FGD1, Program Manager). “Well, I would say there may be a misconception that when they go to the doctor that they—they are gettin’ tested for STDs, and they may not be gettin’ tested. You know? They’re just—they-they do not realize they have to get—um, ask or request that test. You know what I mean?” (FGD4, Case Manager).
Financial limitations	“…if you do not have insurance… that’s gonna prevent you if you wanna come to our clinic. But there is no appointments because we so many patients and not enough money to pay providers to see ‘em. Um, yeah. Just kinda that-the healthcare system as a whole.” (KPI05, Medical Director/Clinician). “I think that there is, um, obviously some issues with accessing. If you do not have private health insurance, you are limited to where you can access PrEP for a low or free cost.” (KPI09, Executive). “And honestly, I think it’s, uh, it’s also—I think sometimes the healthcare community is afraid to test someone if they think they do not have insurance…” (KPI10, Medical Director/Clinician).
Stigma and discrimination	“For the same reason, embarrassment [may keep sexual and gender minorities, from accessing HIV testing services].” (KPI02, Program Manager). “Um, a fear of, you know, what are they gonna—what’s that person gonna think of me? What’s a person gonna—is this person gonna judge me?” (KPI05, Medical Director/Clinician).
Facilitators	
Raising awareness of PrEP efficacy and accessibility through education	“Um, I’ve gotten a lot of questions about if it actually works. Uh, they wanna know if it works, …so probably is it effective…?” (FGD4, Case Manager). “We’ve gotta educate them on that, like, this is free. So we pay for all of the labs in the office visit. We enroll clients in the patient assistance program so there’s no cost to them… and we will provide transportation.” (KPI04, Program Manager). “We-we have a, um—a marketing—community marketing and awareness campaign where we put up billboards… encouraging PrEP uptake around PrEP, helping people to understand that it’s not just for individuals at risk for HIV. It’s not just for [men who have sex with men] MSM, it’s for anybody.” (KPI03, Health Department Executive).
If they are supported financially, they can overcome stigma	“If I do not have insurance and I can get it and it—and it’s not gonna cost me anything, then I’m just gonna do what I need to do, regardless of what the stigma might be around it.” (KPI03, Health Department Executive).
Awareness of local programs that offer financial support for PrEP services	“Um, we have been able to partner with [local university] to offer gift cards to people who come into their first PrEP visit… So, we have-we-we took away that barrier, but in-in some instances, um, where you have a [/an/] income restriction and that kinda thing, that might be a challenge for individuals accessing PrEP.” (KPI04, Program Manager). “One of the good things that we have here in [local] County is the County earmarked a certain amount of dollars, um, to give money to six clinics. And we are one of those clinics that, if you are a resident in [local] County, your care is free for PrEP if you are uninsured or under-insured.” (KPI11, CBO Executive).

**Table 4:** BSGM community member perspectives: Compassion from Others is the Antidote to Mistrust When We Do Not Know Who to Believe.

Summary	Quotes
Participant A shares a “testimony” of feeling judged and unsupported by healthcare providers after seeking PEP following a non-consensual exposure through “stealthing,” or the secretive removal of a condom. In a separate incident, after gallbladder surgery, Participant A faced further judgment from providers who dismissed his health concerns, attributing them to weight.	Participant A “…the first place I went to was Planned Parenthood…. They could not help me, and they-they sent me to the, uh, clinic, uh, the [local] County Clinic. And, um, when I got there, nobody was really helpful, and ev—the-the nurses were asking me… “Well, what’s the big deal if you do not know if this person has HIV or not?” […] nobody was really helpful… [they] sent me to [organization], which is a LGBT place here, and she told me to go to the nearest emergency room to get PEP and that [their organization] would help me cover the cost of it since I did not have insurance. Um, I went to the emergency room. They also had never heard of PEP. […] I’m anxious ‘cause I feel like, you know, that clock is tickin’, takin’ longer and longer. The longer we wait, the less effective it is. Um, so a nurse who I guess happened to overhear […] another gay man… he was a lifesaver… Um, it was a kind of—the-the nurses at the clinic made me feel very stupid. And I felt judged, and it-it made me not want to go back there.” (Cis man, taking PrEP, 36 years) “… I’d experienced that most of my life more so than the Black thing, just the fat thing. If I come into a emergency room or complaining about any kinda ailment, the first thing out their mouth was, “Well, you need to lose weight. I-I-I fell and twisted my ankle, ma’am, me losin’ 100 pounds is not gonna stop that.” (Cis man, taking PrEP 36 years)
Participant B highlights the importance of supportive networks in staying healthy; that sharing experiences and advice with others helps them make better health decisions.	Participant B “…I get all of the support I need… Um, I would say it helps me stay healthy, I mean, ‘cause, you know, sometimes you can-you can think you are doin’ the healthy-a healthy thing or eating right or doin’ the right thing, but then you start talkin’ to other people, and then, you know, you find out things like they done been to a doctor. The doctor said they should be doin’ this. You know, so, they should not be doin’ it, you know, 9 times outta 10, you probably should not be eatin’ that either.” (Cis man, taking PrEP, 39 years)
Participant C discusses the discomfort they feel when doctors label them as “high risk” due to their perceived *identity* as a man who has sex with men, or the unfavorable, “MSM.”	Participant C “Um, I think—I have not been treated poorly. But I think the only thing that comes to mind is, um, when doctors say because, um, because I have so many—when they ask questions about, you know, in reference to HIV and they ask questions about how many partners you have at the time. And I kinda think, and, um, how you could be high risk because of those—things, which is true, but I do not know if I necessarily like the wording of being high risk because, you know, I’m a man that has sex with men—Um, I just try to be very careful. I make sure that the—when I’m either asking for recommendations for a new doctor or someone I’m going to that they are, um, LGBTQIA-friendly, um, and kind of sensitive to that area. So that way I will not, you know, face that discrimination…. just kinda leaning on friends and asking their, um—kind of asking their a—or other people I know who are in the, um, medical field or health field, healthcare field, for recommendations for people to go to.” (Cis man, taking PrEP 35 years)
Participant D describes the crucial role of supportive friends and coworkers who provide emotional, mental, and financial support, reminding them to prioritize self-care. The second quote contrasts this with a negative healthcare experience, where Participant D felt scrutinized and stigmatized when trying to refill their PrEP prescription while recovering from an injury.	Participant D “I have amazing group friends, and amazing group of coworkers—that, um, I share my day-to-day to life with. Um, they are great moral support for anything. Um, it was fun for me. Um, they support me in multiple ways whether it’s just getting an ear to listen to, or when I fall short on certain things whether financial, mentally, they are always there to help support me. So, that’s one thing I can say about the support I get around me… Um, with my lifestyle and with my job, with just being human, sometimes life moves quick. And it’s good to have those reminders from my friends of like to take—make sure I’m taking care of myself…” (Cis man, taking PrEP, 24 years) “Um, I had a bad-a bad experience when I had broke one of my limbs… I was out of the prescription I was sent to the hospital for a couple of days, and I needed someone to refill my PrEP for me. And the hospital would not fill it for me. Instead they’d rather [ask] hundreds of questions of why I’m taking the drug. Instead of getting in contact with my primary care doctor, and having that prescription refilled… Um, I felt as if I was under a microscope…-my prevention and my lifestyle was more glorified…” (Cis man, taking PrEP 24 years)

**Table 5:** Leadership perspectives: Compassion from Others is the Antidote to Mistrust When We Do Not Know Who to Believe.

Barriers	
Providers who are not compassionate or affirming	“I have not, um, encountered it myself, but when I worked at a different clinic, it was shared with me that, uh, one of the physicians, actually, they stopped him from seeing the clients who wanted, um, PrEP or those who were positive for HIV because he was older, um, like 70s, 80s. And so his mindset was not open to today’s world. Um, so he would tell them, you know, “You’re sinning.” [*Laughter*] (FGD2, Case Manager).
Parental mistrust	“So our-our big issue with, um, PrEP has been that our clinic is set up to just see uninsured, uh, patients because we have other providers who have the capacity to see insured patients. So we, you know, decided to serve the uninsured. And what we have found is many people h—when we get to the point where we are signin’ them up for the patient assistance program, have insurance. So a lot of our young people are on their parents’ insurance, and they do not want their parents to know.” (KPI01, Medical Director/Clinician). “When someone says, “Hey, oh, I know I need to be tested,” and even if they already have their results, and they are negative, if they are interested in initiating any type of preventive treatment, the concern, especially with our young people, are, you know, I’m still on my parents’ insurance. I do not want them to know, or maybe I have not even disclosed to them that I—that I am high-risk, and that’s the reason why they do not want their parents to know.” (FGD3, Public Health Investigator).
Knowledge of reasons to take PrEP	“And then also, you know? For Black MSM, it’s [sighs] a lot of the times it’s just, like, they are like, ‘Well, I could get on PrEP. But I think I can-I think I can avoid-I think I can avoid it.’ And, you know, ‘I can scope—I can scout people out.’ I have noticed that a lot of Black MSM men who I interview either for—you know? They contracted syphilis, gonorrhea, chlamydia, anything like that—they are like, ‘Oh, no. I do not mess-I do not mess around. I do not be on Grindr. I do not be on the apps. I keep it—I’m one—I’m only one person. I’m not like them other—I’m like my other people in my community.’” (FGD1, Public Health Investigator).
Reliance on partner trust/Knowledge of reasons to take PrEP	“We-we have individuals who will get into a monogamous relationship, or they feel that it’s a monogamous relationship, and stop taking PrEP, um, because they feel like there’s no need for them to take it… I think I’m going back to this knowledge thing, information, education.” (KPI11, CBO Executive).
Double-edged sword of representation	“I think sometimes we hire from within the community to make sure that there’s representation, but the community is so small that sometimes there’s, like, one degree of difference. So, like, my roommate might have dated somebody that works at that facility. Or, um, and I think that that can be intimidating. Or, um, they may think that, you know, because they are in the community, that their status is gonna get out if they are positive.” (KPI09, Executive). “It’s a population that I wish we saw more representation here because I, um, I imagine they are out there and they are just not seeking, um, healthcare opportunities because of, you know, a lot of reasons, including not being as trans- friendly as I wish we were.” (KPI05, Medical Director/Clinician).
Facilitators	
Representation builds trust	“Trans individuals, a lot of them that I see, they are only interested in my opinion and in my personal experience ‘cause I-I have had many trans clients.” (FGD1, Program Manager). “I wish that we had, you know, just better representation. Even like a staff member that was trans. I mean that’s a little bit harder. But I think that peer-to-peer, um, creates an environment that’s a bit more comfortable.” (KPI05, Medical Director/Clinician). “I mean, I think-I think it’s been more important [having individuals who identify in these communities] … when it comes to our educational efforts and just, uh, awareness efforts.” (KPI06, Program Manager).
Not trusting sex partners	“Some patients, even though now, like, um, I’m gonna talk about-I’m gonna talk about cis-women just for a second… ‘That man really was cheating on me, and he could be having sex with so-and-so and so-and-so. And if I’m gonna keep having sex with him, and if these men are gonna keep doing x, y, and z for me, then yeah. I might as well-I might as well take some PrEP.’” (FGD1, Public Health Investigator).

#### Theme 1: Navigating and Negotiating PrEP: “Survival” vs. “Health”

Leadership participants and BSGM community members highlighted the tension between survival and accessing PrEP services for BSGM. “Survival” in this context refers to meeting basic survival needs, including employment, financial stability, general education, and access to food and transportation. Participants emphasized the need for comprehensive healthcare, which may include social services such as employment assistance and other economic mobility drivers like educational and medical resource provision. Once able to access prevention services, BSGM participants listed challenges such as lack of privacy and discretion when receiving PrEP medication and discomfort with identifiable medication packaging.

##### Leadership perspectives

The experiences of BSGM community members align closely with leadership perspectives, reinforcing the systemic and individual-level barriers that hinder access to HIV prevention services from both perspectives. Leadership participants also highlighted socioeconomic barriers such as housing instability, food insecurity, and education (i.e., lack of awareness regarding PrEP access). Both groups emphasized the importance of having readily available and clear information about PrEP programs to help overcome these barriers.

#### Theme 2: Compassion from Others is the Antidote to Mistrust When We Do Not Know Who to Believe

Participants across data sources described the complex role of trust in accessing HIV prevention services. BSGM community members revealed that trust in healthcare providers and within social networks may be essential for engaging with HIV prevention. Though not always related to seeking HIV services, BSGM described significant barriers resulting from negative experiences with healthcare providers, where biases and lack of understanding lead to poor treatment and a deep mistrust in the medical system. Supportive relationships with peers and providers, accurate information, and finding empathetic, lesbian, gay, bisexual, transgender, and queer plus (LGBTQ+) friendly healthcare providers were identified as crucial facilitators for overcoming these barriers.

##### Leadership perspectives

Leadership participants reflected similar concerns about trust and the dissemination of accurate information within BSGM communities. Trust in providers, particularly those with shared lived experiences, may enhance engagement with services. Conversely, a lack of trust or misinformation can lead to reduced uptake of preventive services. Participants emphasized the need for compassionate care and trustworthy relationships with providers to overcome these barriers and encourage consistent use of HIV prevention services. Leadership participants shared concerns related to medical mistrust and lack of provider compassion, similar to experiences shared by BSGM community members.

## Discussion

This study addresses critical gaps in HIV prevention for BSGM communities in the US South, where social and structural barriers exacerbate health disparities. Our findings reveal two key themes: (1) How do We Navigate and Negotiate PrEP when the Options are “Survival” vs. “Health”? and (2) Compassion from Others is the Antidote to Mistrust When We Do not Know Who to Believe.

Within the first theme, BSGM perspectives reveal a tension between accessing HIV prevention services and satisfying basic needs for stable housing and food. Participants described how a lack of education and access to health information and limited financial resources hinder prioritizing HIV prevention or “health.” Additionally, leadership and BSGM community members expressed concerns about confidentiality, fears of stigmatization, and mistrust of healthcare providers, which further impede accessing services like PrEP. Together, these findings underscore how clear, accessible information about PrEP and discreet service delivery options can foster a supportive, safe(r), stigma-reduced environment for BSGM individuals seeking HIV prevention services.

Within the second theme, BSGM community members shared negative experiences, spanning judgmental treatment, feelings of being unsupported, and directly dismissive attitudes from health care providers, resulting in tangible delays in care and mistreatment. These encounters were shared as additional drivers of mistrust in the healthcare system, further exacerbating a series of discouraging HIV prevention engagement experiences for BSGM. Conversely, participants identified how trusted, compassionate, LGBTQIA-friendly providers, and empathetic support from social networks are vital to alleviate stigma and medical mistrust. BSGM community members highlighted instances where compassionate interventions—such as guidance from peers or LGBTQIA-friendly healthcare providers—helped restore trust and encouraged proactive health behaviors. Collectively, study insights speak to the urgent need for equitable, dignified, and compassionate care tailored to the basic human rights and needs of BSGM individuals. Without compassionate care, the route to informing and supporting BSGM is unclear, and likely remains a major pitfall of access to HIV prevention services.

These findings support and expand upon existing literature on BSGM engagement in HIV prevention services, underscoring how structural and social determinants, particularly in the US South, exacerbate healthcare disparities (2). They reveal the unique barriers BSGM individuals face, often compounded by intersecting issues of stigma, socioeconomic disadvantage, and discrimination. Addressing these barriers necessitates tailored, locally responsive interventions, especially for transgender women and other underrepresented groups who face additional structural challenges in accessing PrEP and related services (4, 32, 33).

For instance, we know that social networks play a pivotal role in bridging gaps in healthcare by providing trusted, peer-driven guidance and emotional support, which can mitigate experiences of stigma and mistrust (34). Participants highlighted the value of community and social networks in encouraging HIV prevention engagement and providing a source of accurate, non-judgmental health information. Social networks may help to overcome systemic mistrust that arises from negative healthcare experiences, especially where provider biases and lack of LGBTQIA+ sensitivity have led to disengagement and harm from care.

Additionally, across the two themes, stigma drove barriers to prevention services uptake: participants’ internal struggles, compounded by anticipated or experienced judgment from providers, often resulted in delayed care or avoidance of services. These results confirm existing literature that highlights the multifaceted nature of stigma and its detrimental effects on health outcomes (2, 3). The driving force of stigma imbues the need for initiatives that reduce stigma, enhance health literacy, and empower BSGM individuals through education and self-advocacy.

Lastly, to foster long-term engagement in HIV prevention, programs are needed that engage and empower BSGM where they are. Study results will be used to develop an enhanced Social Network Strategy (eSNS) to leverage social networks to disseminate accurate, culturally responsive health information and bolster community support. Social network interventions may be critical to increase PrEP uptake and inform how we can create more positive experiences within HIV preventive healthcare. Through eSNS, the power of trusted social networks may counter stigma, rebuild trust in healthcare systems, and enhance BSGM access to HIV prevention services. Consistent with qualitative inquiry principles, our findings emphasize transferability rather than generalizability, offering insights that may apply to similar sociocultural contexts within and beyond the US South.

### Limitations

While the study provides valuable insights from leadership perspectives and BSGM community members, future research incorporating diverse viewpoints, including those from more than four community members, and including transgender women, is warranted to ensure a comprehensive understanding of HIV prevention service engagement among BSGM populations. Despite efforts to foster open dialog, the possibility of groupthink in focus groups—a phenomenon where the desire for conformity leads to the suppression of dissenting opinions—cannot be entirely discounted (35). However, rigorous interview techniques and facilitation aimed at promoting diverse perspectives were employed to mitigate this potential bias. The multi-site nature of the research team may also have implications for understanding local contexts. To address this limitation, follow-up interviews post-implementation are planned to expand findings and enhance data validity.

It is also important to discern the meaning of emerging themes alongside social network theory. Although the BSGM community member interview guide was informed by social network theory, participants rarely described their experiences using explicit “network” language. Instead, they emphasized trust, representation, and compassion, which are relational elements that operate as the social mechanisms within networks. This pattern may reflect how BSGM participants conceptualize connection less as a structural network and more as relational trust grounded in shared experience and community care. These insights inform eSNS by placing focus on relational trust and compassion as the foundation of effective network-based HIV prevention strategies.

Lastly, this work does not address more structural issues (i.e., policy) identified by both leadership and community members. Rather, the study’s purpose was to identify barriers and facilitators that will guide the design of the forthcoming eSNS intervention; and, while our findings are situated within the Southern context, similar dynamics may manifest in other regions where systemic inequities, stigma, or limited access to affirming care persist. Our findings involve both contextual specificity and potential transferability. Future studies should focus on strategies to address barriers rooted in structural policies and advocate for reforms to eliminate discriminatory practices and promote inclusive healthcare environments.

## Conclusion

Our qualitative study explored HIV prevention services for BSGM groups in the US South from the perspectives of HIV service leadership and members of the community. Participants indicated that without basic living needs met, HIV prevention remains a lower priority among BSGM. If BSGM can access HIV prevention services, it is important that they are met with compassion, which necessitates interventions that identify and address modifiable barriers and actionable facilitators. These findings will inform an eSNS intervention tailored with inclusive messaging, local service information, PrEP modalities, and stigma reduction strategies to bolster HIV prevention among BSGM.

First, the findings illustrate the need for wraparound services that address living necessities as a determinant of accessing HIV preventive care. Second, compassion and empathy are synonymous with ethical and responsible approaches to HIV prevention and are critical to supporting BSGM and reducing harm. Finally, our findings illustrate that stigma is a pervasive barrier at every level of the HIV prevention continuum, significantly impacting the health and wellbeing of individuals living with and at increased chance of acquiring HIV. By tackling stigma across levels, it is possible to create an environment where individuals feel safe and supported in seeking the health services they need, ultimately improving health outcomes and quality of life for anyone whose lives may be touched by HIV.

## Data Availability

The datasets analyzed in this study contain sensitive qualitative information. Participants did not consent to public sharing. Data may be available upon reasonable request to the corresponding author, subject to ethical approval.
