# Supplementary material for: Survival, trust, and compassion: persistent HIV prevention concerns faced by Black sexual and gender minority communities in the US South – a qualitative study
Source: Front Public Health. 2025 Nov 14;13:1695474. doi: 10.3389/fpubh.2025.1695474 (PMC12660203; doi:10.3389/fpubh.2025.1695474)
Supplement: Supplementary file 1 [file Data_Sheet_1.PDF]

Table S1. HIV Prevention Interview Questions

In-depth Interview (IDI) Questions	Focus Group Discussion (FGD) Questions
• What do you think are some of the things that may keep some **sexual and gender minority groups** from accessing **HIV testing services** at your facility?	• What do you think are some of the things that may keep some **sexual and gender minority groups** from accessing **HIV testing services**?
• What do you think are some common things that may keep **Black men who have sex with men** from accessing **HIV testing services** at your facility?	• *What do you think is most important to **Black men who have sex with men** when accessing **HIV testing services**?*
• What do you think are some common things that may keep **transgender women** from accessing getting **HIV testing services** at your facility?	• *How do these compare among **Black transgender women**?*
• What do you think are some of the reasons some **sexual and gender minority groups** may not be getting HIV prevention services like **PrEP** at your facility?	• What do you think are some of the reasons some **sexual and gender minority groups** may not be getting HIV prevention services like **PrEP**?
• What do you think are some common things that may keep **Black men who have sex with men** from accessing HIV prevention services like **PrEP** at your facility?	• *What do you think is most important to **Black men who have sex with men** when accessing HIV prevention services like **PrEP**?*
• What do you think are some common things that may keep **transgender women** from accessing getting HIV prevention services like **PrEP** at your facility?	• *How do these compare among **Black transgender women**?*

Table S2. Example questions from BSGM network members in depth interviews

**HIV Services**	1. What is your experience getting tested for HIV?<br>2. As you may know, pre-exposure prophylaxis or PrEP is a pill that you can take to prevent HIV. It is also available now in a long-acting injectable form. Tell me any thoughts you have about the option of using PrEP to keep from getting HIV?<br>3. Tell me about any experiences you have had seeking and/or receiving HIV prevention programs, such as PrEP?
**PrEP**	1. How do you feel about disclosing that you are currently taking PrEP to others, such as your partner, family, relatives, etc.?<br>2. What are some of the challenges you have in receiving PrEP?<br>3. What are some of the things that make it easier for you to receive PrEP?<br>4. Would you recommend PrEP to your friends who you think may benefit from it?<br>5. What do you think we should consider to help more people access PrEP programs?
